# Supplementary material for: Shared Treg dysregulation underlies psoriasis vulgaris and atopic dermatitis through the PHF19-PRC2 epigenetic axis
Source: Front Immunol. 2026 Aug 20;17:1932788. doi: 10.3389/fimmu.2026.1932788 (PMC13538097; doi:10.3389/fimmu.2026.1932788)
Supplement: Supplementary file 3 [file DataSheet3.zip › Additional files/Additional file 7/11.B4GALT1.funnel.pdf]

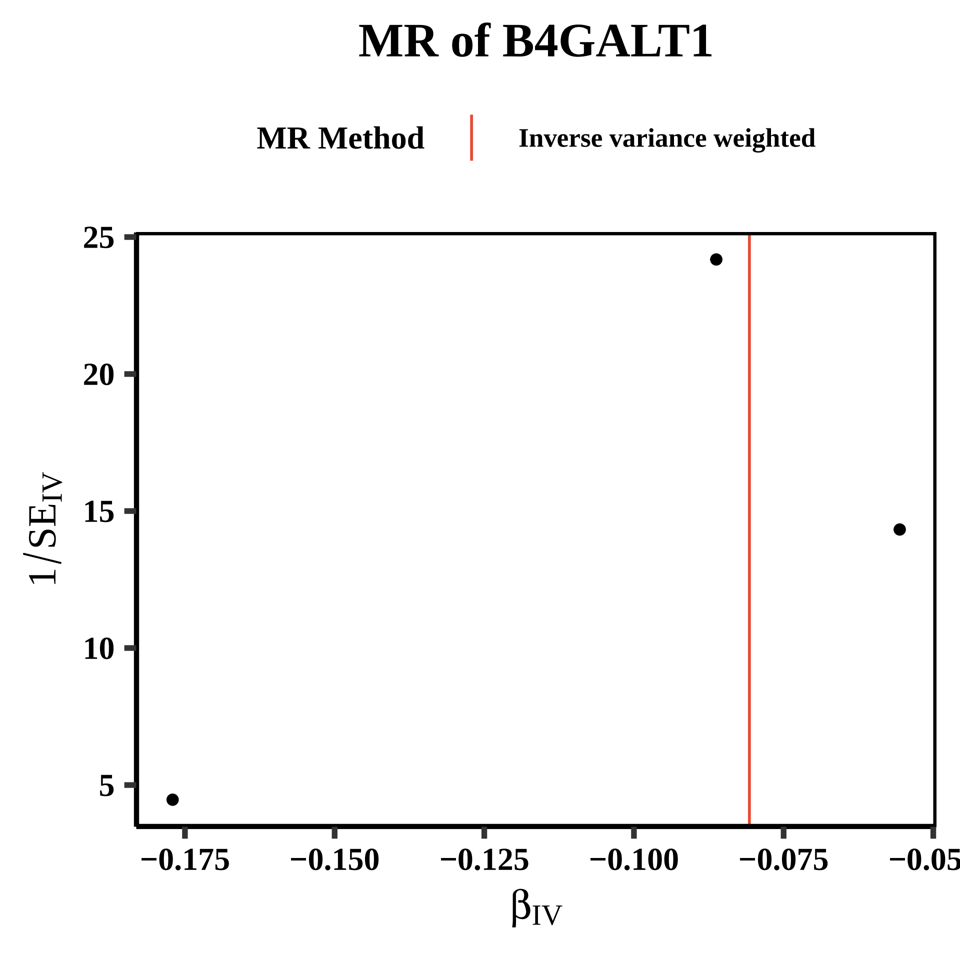

MR of B4GALT1
MR Method
Inverse variance weighted
25
20
15
10
5
1/SE$_{IV}$
−0.175
−0.150
−0.125
−0.100
−0.075
−0.05
β$_{IV}$
